# Kinetic changes of intestinal microbiota in the course of intestinal sensitization

**DOI:** 10.18632/oncotarget.12797

**Published:** 2016-10-21

**Authors:** Liang Xiao, Bo Yang, Xiaoyu Liu, Yan Luo, Qiongmei Ji, Zhong Wen, Zhigang Liu, Ping-Chang Yang

**Affiliations:** ^1^ The Research Center of Allergy and Immunology, Shenzhen University School of Medicine, Shenzhen 518060, China; ^2^ BGI Shenzhen, Shenzhen 518000, China; ^3^ Department of Otolaryngology, Zhujiang Hospital, Southern Medical University, Guangzhou 510280, China; ^4^ Key Laboratory of Optoelectronic Devices and Systems of Ministry of Education and Guangdong Province, College of Optoelectronic Engineering, Shenzhen University, Shenzhen 518060, China

**Keywords:** food allergy, intestine, microbiota, 16S rRNA, animal model

## Abstract

Food allergy (FA) is an adverse immune response to certain innocent food. It is estimated about 2% to 6% of the general population suffer from FA. Symptoms of a food allergic reaction may involve the gastrointestinal tract or/and other organs. The gut microbiota plays a critical role in diet-induced health problems. Whether the changes in the composition of the intestinal microbiota regulate allergic responses to food remains poorly understood. Thus, we created an FA animal model, sequenced the V4-V5 regions of 16S rRNA genes to characterize the genera abundance of gut microbiota. The results showed that mice under FA condition showed different gut bacterial structures. Diverse distribution of the bacterial species was identified between FA and control groups. FA altered the components of intestinal Microbiota in mice. The dysbiosis of the gut metagenome correlated with the development of the FA.

## INTRODUCTION

Food allergy (FA) is an IgE-mediated allergic response in the body; the pathogenesis if unclear. The symptoms of FA mainly occur in the intestine; but also occur in other organs, such as in the skin, airway and kidney. The immune pathological features of FA is the over expression of T helper (Th)2 cytokines, including interleukin (IL)-4, IL-5 and IL-13, etc, which can be detected in the sera and in the local tissue. Although the research in FA was advanced rapidly in the recent years, the mechanism of Th2 polarization is still to be further investigated.

Several FA mouse models have been published. Among the materials used in the development of FA model, microbial products, such as cholera toxin, Staphylococcal enterotoxin B, were used as adjuvants in the sensitization. The fact implicate that the alteration of amounts of microbes or the composition may be related to the pathogenesis of FA; this is to be further investigated.

As our understanding of the profound influence of commensal microbes on the maturation of the immune system has grown, more recent iterations of this hypothesis have supported the idea that alterations in the composition of the intestinal microbiota induced by environmental factors (e.g., antibiotics, diet, vaccination, sanitation) play a central role in the regulation of allergic sensitization [1]. A microarray analysis of intestinal epithelial cells of gnotobiotic mice revealed a mechanism that Clostridia instruct immune cells to produce interleukin-22 (IL-22), regulate innate lymphoid cell functions and intestinal epithelial permeability to protect against allergen sensitization [2]. Gut microbiota stimulate the mucosal immune system through the maturation of the gut-associated lymphoid tissue, which occurs predominantly in the small Intestine. Further evidence needs to be added to demonstrate how the microbiota and immune system can interact to maintain homeostasis. To confirm the different components of the intestinal microbiota, the next-generation sequencing method was used to detect the 16S rRNA in feces samples and to determine the frequency of microbes and its metabolic pathways in the gastrointestinal tract.

Based on the above information, we hypothesize that the sensitization may alter the composition of intestinal microbes. To test this, we developed an FA mouse model. The microbes in the feces were screened by analysis of the 16S rRNA approach. The results showed that the sensitization indeed altered the composition of intestinal microbes.

## RESULTS

### Development of a food allergy (FA) mouse model

**Figure 1:**
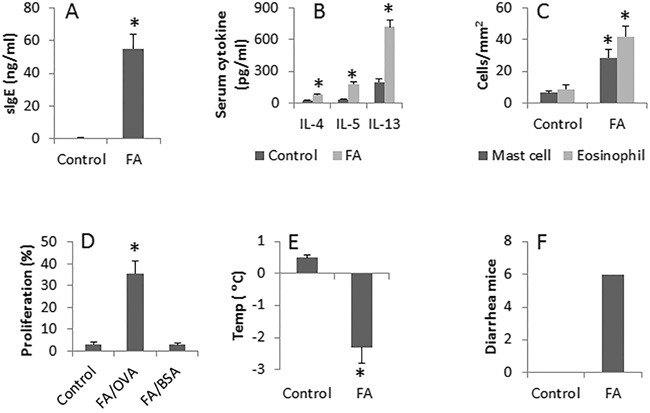
Assessment of allergic inflammation in the mouse intestine BALB/c mice were sensitized to ovalbumin with cholera toxin as an adjuvant. Control mice were treated with saline. The bars indicate the serum levels of specific IgE (sIgE) (**A.**; by ELISA), serum Th2 cytokines (**B.**; by ELISA), the cell counts of mast cells and eosinophils in the intestinal mucosa (**C.**, by histology), OVA-specific CD4^+^ T cell proliferation (**D.**, by flow cytometry), the core temperature (Temp) drop **E.** and the mice with diarrhea upon the specific antigen challenge **F.** Each group consists of 6 mice. Samples from individual mice were analyzed separately. Control: Control group. FA: FA group.

Following our established procedures [3], we developed an FA mouse model. The results showed that the serum levels of specific IgE (Figure 1A, Supplementary Table S2) and Th2 cytokines (including IL-4, IL-5 and IL-13) (Figure 1B) were increased significantly as compared to the control (CK) mice. We also found that abundant infiltration of mast cells and eosinophils in the intestinal mucosa of the FA group than that of the CK group (Figure 1C). To assess the antigen-specific T cells in the intestinal mucosa, we isolated CD4^+^ T cells from the mouse intestinal mucosa and analyzed by flow cytometry. The results showed that about 38.5% antigen CD4^+^ T cells were detected upon the stimulation of OVA (the specific antigen) in the culture, which was not detected in the CK group (Figure 1D). In addition, after challenge with OVA, the FA mice showed the core temperature drop (Figure 1E) and diarrhea (Figure 1F), which did not occur in the CK group. The results indicate that the allergic inflammation was developed in the mouse intestine.

### Mice with FA have different gut bacterial structures from control mice

#### The alpha and beta diversities change

**Figure 2:**
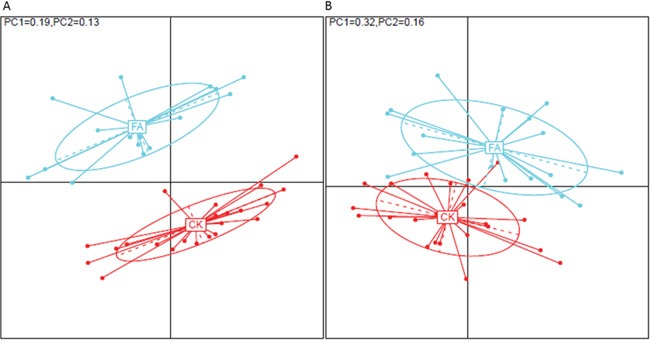
The PCoA based on A. OTU profile and B. genus profile between CK and FA group The samples from control group (red dots) and from FA group (green dots) show a clear separation on both OTU and genus levels, demonstrating an obviously different in the bacterial species structure of gut microbiota under a FA condition. The contribution of Principal coordinate 1 (PC1, x axis) and 2 (PC2, y axis) are shown on upper left.

The principal coordinates analysis (PCoA) [4] were performed based on the OTU and genus profiles of all the samples. The FA group showed a clearly separate from the samples in the CK group, demonstrating that gut microbiota of mice under the FA is significantly different from that in the CK group. The result of the PERMANOVA test [5] also demonstrated that the factor “allergy” owned a significant effect on the gut microbiota of samples at both OTU and genus levels (p < 0.001, Figure 2).

**Figure 3:**
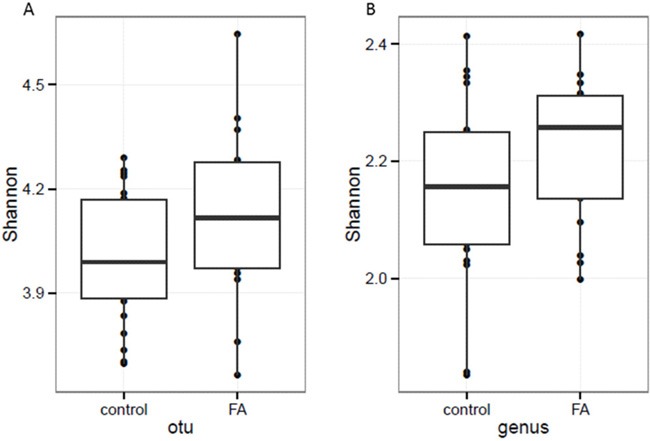
The α-diversity assessed with Shannon index based on A. OTU and B. genus profiles At both OTU and genus levels, the FA group (n=17) showed higher α-diversity than CK groups (n=22), but not significantly (p value = 0.09 and 0.08 respectively).

The alternation of the gut microbiota diversity was reported to correlate with some of the diseases [6]. Thus, we also assessed the changes of alpha-diversity between CK and FA groups. The Shannon index [7] of FA group was higher than of CK, but showed no significant difference (p > 0.05) at OTU and genus levels (Figure 3).

### The distribution of the bacterial species in FA and CK groups

**Figure 4:**
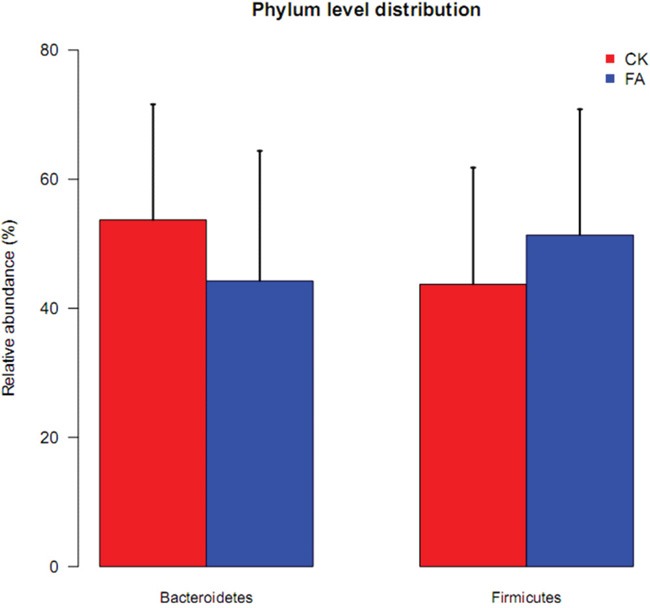
The relative abundance of *Bacteroidetes* (left) and *Firmicutes* (right) in CK (red) and FA (blue) groups The relative abundance of *Bacteroidetes* was decreased, while the *Firmicutes* was increased under a FA condition. The abundance changes of the main bacterial phyla illustrated the perturbation of allergy disease on gut microbiota. Data are presented as mean ± SD.

**Figure 5:**
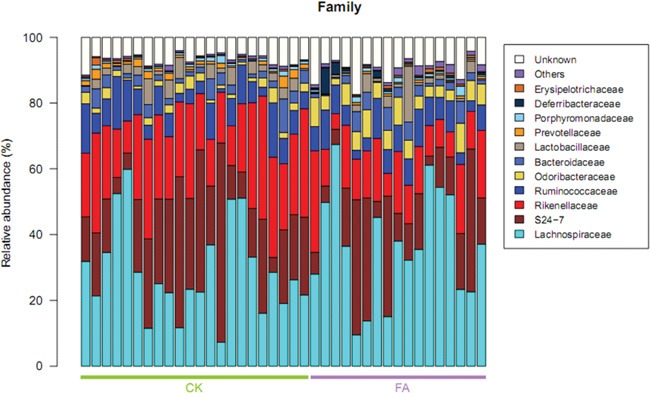
Taxonomic distribution of bacterial families in all samples (n=22 in CK group, n=17 in FA group) The *Rikenellaceae* and *Prevotellaceae* were enriched in CK group, while the relavtive abundance of *Deferribacteraceae* and *Lachnospiraceae* was increased in FA group, demonstrating that gut microbiota is changed at family level under FA condition. Values represent the relative abundance across all samples.

The taxonomic structure was calculated based on the annotation and relative abundance of OTUs. Taxa was assigned using the Greengenes Database [8]. The *Bacteroidetes* and *Firmicutes* are two major bacterial phyla of the gut microbiota. The ratio of *Bacteroidetes*/*Firmicutes* was correlated with the development of obesity and some other diseases, according to previous studies [9]. The relative abundance of the *Bacteroidetes* was decreased under the allergy condition while the *Firmicutes* was increased (Figure 4). The average ratio of *Bacteroidetes*/*Firmicutes* was 1.73 in FA group and 1.26 in the control group. The change of the ratio illustrated the perturbation of the gut microbiota under the FA condition. The tags were annotated to the family level, so the distribution of gut bacterial families was also shown in Figure 5, the *Lachnospiraceae*, *S24-7*, *Rikenellaceae*, *Ruminococcaceae*, *Lactobacillaceae* account for most of family components.

### FA alters the components of intestinal microbiota in mice

**Figure 6:**
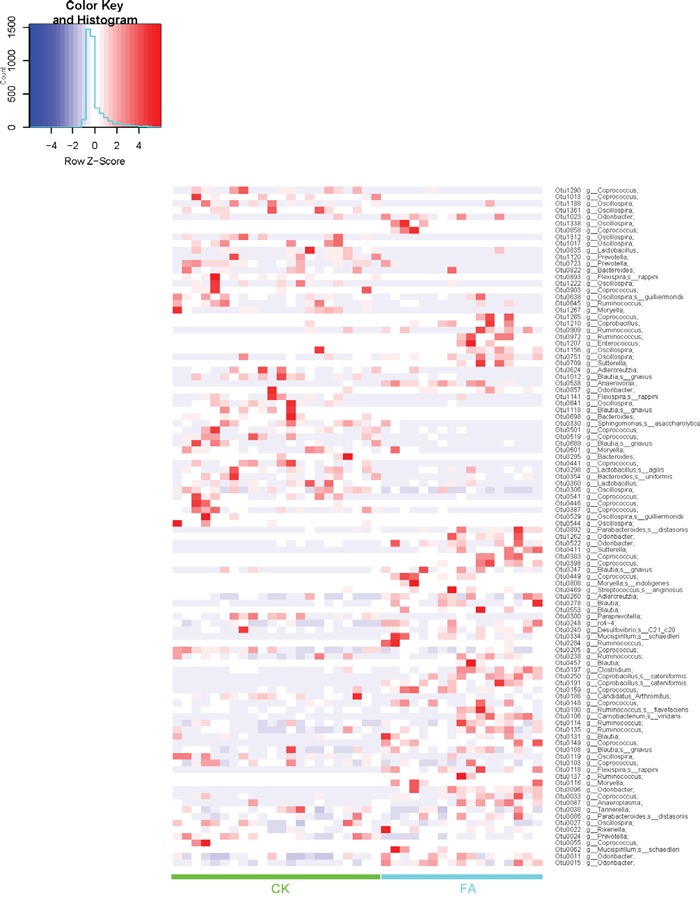
Heatmap demonstrating relative abundance of OTUs in all samples from the CK (n=22) and the FA (n=17) group Columns depict individual mice. The annotation of OTU follows OTU IDs, only OTUs at genus (g_) or species (s_) level are picked out. Different OTUs were enriched in CK and FA groups respectively. The influence of FA condition on the gut microbiota was shown at bacterial species level.

**Figure 7:**
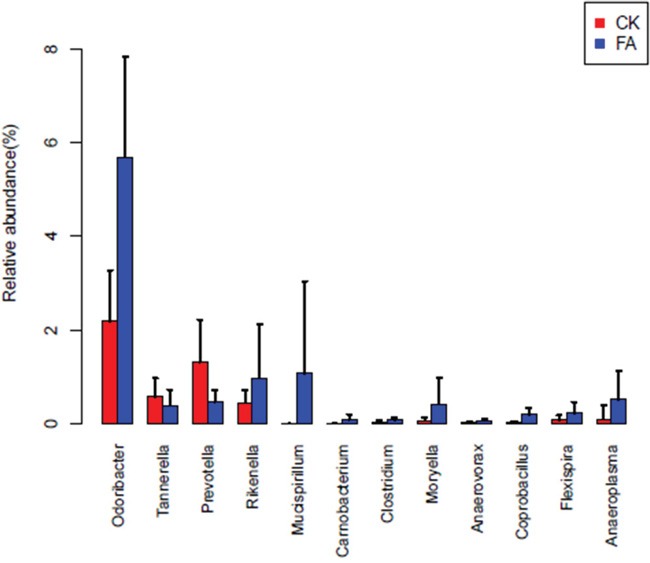
The distribution of 12 bacterial genera in CK (red) and FA (blue) groups The barplosts show the relative abundances among the bacterial genera (p<0.05, FDR=0.124). The *Odoribacter*, *Rikenella*, *Moryella* and *Anaeroplasma* were enriched in the FA group. Especially the genus *Mucispirillum*, it was almost absent in the CK group but increased to more than 1% in the FA group. Error bars show standard error of the mean (SEM).

To explore the changes of gut bacterial species under the allergy condition. The Wilcox sum-rank test was applied to the OTU and genus profiles. The bacterial OTU and genera, which showed the significantly different relative abundance between CK and FA groups, were tested out. At the OTU level, 365 OTUs are significantly different between CK group and FA group (Wilcoxon rank test at the OTU level, p <0.05, FDR=0. 183, Supplementary Table S3), of which 102 OTUs could be annotated to genus or species levels. The heatmap (Figure 6) showed the enrichment of these OTUs in CK or FA groups. *Parabacteroides distasonis* (OTU0892), enriched in the FA group, was reported predominating in the intestinal inflammation [10]. The *Flexispira rappini* (OTU0118) were reported introducing IBD in mice [11], were also enriched in the FA group. Furthermore, at the genus level, the *Mucispirillum*, enriched in the FA group, have been pointed to its opportunistic nature given its putative capacity to degrade mucin and cause colitis by a previous report [12]. Furthermore, the Odoribacter, Rikenella, Moryella etc. bacterial genera were also enriched in FA groups (Figure 7).

In the analysis above, we compared the taxonomic structure of the mouse gut microbiota between CK and FA groups. The results demonstrated that the gut microbiota was changed tremendously under FA condition, including the increasing of the diversity, the changes in the bacterial species distribution and the enrichment of some bacterial genera. We concluded that the FA condition is correlated with the alternation of the components of intestinal Microbiota in mice.

#### Metabolic pathways are altered in FA mouse

PICRUSt [13] was applied to predict the KEGG orthology (KO) from 16S sequencing data. The KO profile, including 6,909 Kos, was generated based on the relative abundance of the OTUs. Totally 427 KOs were found significantly different in CK and FA groups using Wilcoxon rank test (BH-adjusted *p* value < 0.05), 253 (59.2%) of them are enriched in FA group (Supplementary Table S4). The manganese/zinc/iron transport system, multicomponent Na^+^: H^+^ antiporter, sensor histidine kinase, sulfite reductase, type IV pilus assembly protein, MSHA biogenesis etc. functions were higher, while the AI-2 transport system, xanthine dehydrogenase, the general secretion pathway was lower in the FA group. The KEGG pathways were also generated based on the KO profile and 12 different pathways between CK and FA group were tested out with the Z-score method [14] (Supplementary Table S5). Notably, the N-glycan biosynthesis enriched in the FA group demonstrated that immunoglobulin G molecules increased under FA condition, because all IgG molecules carry *N*-glycans, which modulate their biological activity.

### The correlation between the dysbiosis of the gut metagenome and the development of the FA

#### The bacterial species increase/decrease along with the development of the FA

**Figure 8:**
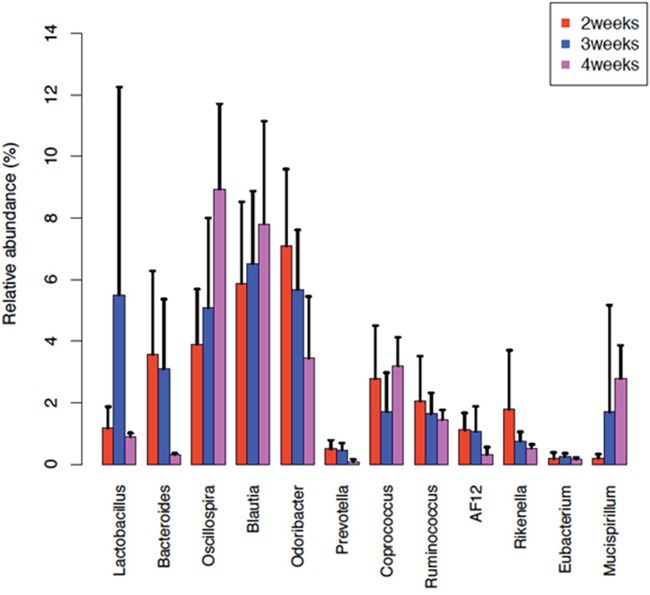
The change of some bacterial genera's abundances along with time points The barplots show the relative abundance of some bacterial genera at 3 time points, 2^nd^ week (red), 3^rd^ week (blue) and 4^th^ week (purple). The relative abundance of *Oscillospira* and *Blautia* was increased along with the FA development. While the relative abundance of *Bacteroides*, *Odoribacter*, *Rikenella* and *Ruminococcus* was decreased along the 3 time points. Error bars show standard error of the mean (SEM).

We selected the samples of the 4 time points in FA group and detected the different OTUs to figure out the dynamic changes of intestinal microflora. The Genus level distribution is also obtained and we discover that the relative abundance of the *Bacteroidaceae*, *Rikenellaceae*, *Odoribacteraceae* decreased, while the Oscillospira, Blautia, Mucispirillum increased in the first 3 time points. These bacterial genera changed along with the development of the FA, implicating that they may closely relate to this disease condition (Figure 8).

#### The network of the bacterial species enriched in FA and CK groups, respectively

**Figure 9:**
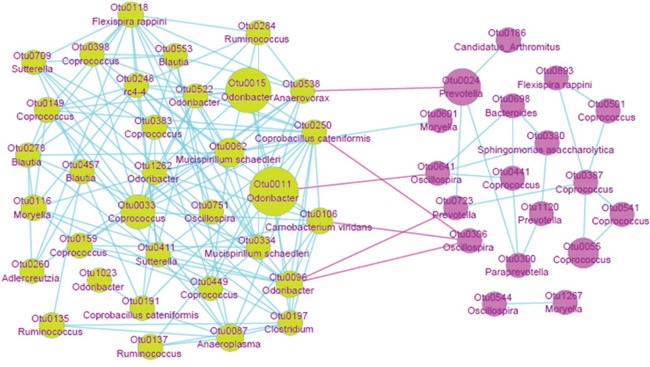
The network of OTUs are enriched in FA (green) and CK (pink) groups Each node represents one OTU, the area of node represents the relative abundance of OTU, the annotations of OTUs are presented under the OTU IDs. Correlation among OTUs enriched in FA group showed much more complication than CK group. OTU0538, OTU0250, OTU0011, OTU0106 and OTU0096 were positively correlated (blue edges, Pearson correlation > 0.4) with other OTUs enriched in FA group, while negatively correlated (pink edges, Pearson correlation < -0.4) with the OTUs enriched in CK group. These bacterial species may play an important role in development of FA.

We picked out 54 significantly different OTUs between CK and FA groups that are on genus or species level, and constructed the interaction network (Supplementary Table S6 & Figure 9). The FA network is obviously more complicated than the CK network, demonstrating more interactions among the gut bacterial species under the allergy condition. The Odoribacter (OTU0011), Clostridium (OTU0197), Coprobacillus cateniformis (OTU0250), Camobacterium viridans (OTU0106) showed more positively coefficient with the other bacterial genera in the FA group, implicating these OTUs play a core role in the gut microbiota under the FA condition. The Oscillospira (OTU0366), enriched in the CK group, was reported specializing in fermenting complex (plant) fiber and supply the products to be used by the butyrate producing bacteria [15]. It is also negatively coefficient with some core bacterial species enriched in the FA group, suggesting that this bacterium may own the potential ability to intervene in the gut microbiota under the FA condition, which may even improve the disease condition.

#### Association analysis of OTU profile and mice phenotype on immune aspects

We measured five immune indices: sIgE, sIgG1, IL-4, IL-5 and IL-13. Through the association analysis of OTU and these five phenotypes. We found some OTUs are closely associated with these phenotypes (*p*<0.05) that were detected with the Spearman correlation coefficient (>0.8). The result showed that totally 15 OTUs could be annotated to species level and correlated with these immune indices (Supplementary Table S7).

**Figure 10:**
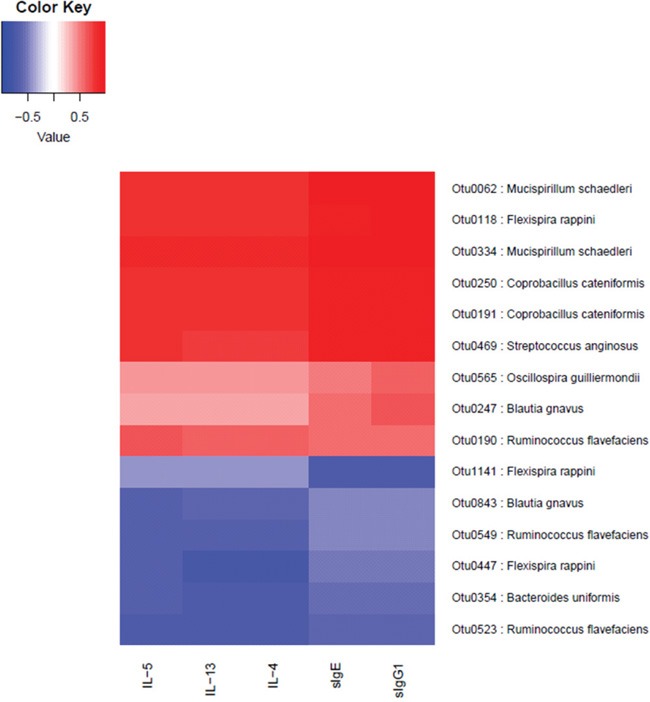
The correlation between immune indices and OTUs The OTUs positively (red) or negatively (blue) correlating (p value < 0.05) with the immune indices were listed on the right with annotations. The correlations between OTUs and immune indices were highly consistent. One OTU positively correlated with one immune index, it was also positively correlated with other indices. Furthermore, two of the OTUs that are positively correlated with all immune indices are annotated as *Mucispinrillum schaedleri* and the other two of them are annotated as *Coprobacillus cateniformis*, implicating that these two bacterial species may closely associate with the immune phenotype changes in the development of FA.

It has to be mentioned that Otu0190, Otu0523, Otu0549 are annotated to the same species *Ruminococcus flavefaciens*, Otu0190 is positive correlated with IL-5, but Otu0523 and Otu0549 are negatively correlated with IL-4, IL-5, IL-13 (Figure 10). This may due to the inaccuracy of Otus annotating to its species. The corresponding bacteria of these OTUs probably involve the immune regulatory network.

## DISCUSSION

In this study, we observed that FA mice showed altered gut bacterial structures. The distribution of the bacterial species was diverse between FA and control mice. The data indicate that FA altered the components of intestinal Microbiota in mice. The resulted dysbiosis of the gut metagenome is correlated with the development of the FA.

The previous studies suggested that host-microbe interactions are critical for inducing motivational signals for the naïve immune system [16]. Healthy gut microbiota may have a crucial role in the maturation of the immune system to nonallergic mod [1]. Thus, microbiota plays an important role in the development and calibration of host immunity, balanced interactions between microbiota and host tissue compartments is necessary and the perturbations in host-microbiota crosstalk may cause allergy and chronic inflammation [17].

The statistical analysis showed no significant differences in the overall bacterial diversity of the gut microbiota between FA and CK groups, it is consistent with the previous study [18]. While the PCoA analysis showed a clear separation in the samples under FA and CK conditions, demonstrating that the distribution of the gut bacteria under FA condition was altered tremendously. To explore it in detail, we use the statistical method to find out the bacterial species changed significantly in the FA samples. The *Parabacteroides distasonis* (OTU0892) enriched in the FA group and the relative abundance of the *Flexispira rappini* (OTU0118), reported inducing inflammatory bowel disease (IBD) in mice [10], was significantly higher in the FA group. Both bacterial species related to inflammation development, implicating a correlation between FA and gut inflammation. We collected the mouse fecal samples of the FA groups at 4 time points to follow the continuous alternation of the gut microbiota along with the development of FA. To one of the bacterial genera, *Mucispirillum*, attention should be paid. This genus enriched in the FA group at relative abundance, while almost absent in CK group. Furthermore, the relative abundance of the *Mucispirillum* increased continuously at the 3 time points of the FA developing. Obviously, this bacterial genus correlated with the FA very closely. According to the published data [19], the *Mucispirillum* own the putative capacity to degrade mucin and cause acute colitis.

The definite fault of previous 16S studies about the gut microbiota is lack of the information at the function level. In the present study, we employed the PICRUSt software to predict KEGG orthology (KO) from 16S sequencing data and investigate the function pathway enriched in FA groups. Lipopolysaccharide (LPS), also known as endotoxin, is found in the outer membrane of the Gram-negative bacteria, which elicits strong immune response. The LPS_biosynthesis module (M00080) enriched in the samples in FA group, according to the results of the present study, illustrate that the LPS originates from gut bacteria may be involved in the development of the FA. Notably, the modules of Sulfur_reduction (M00176) and Nitrogen_fixation (M00175) were also found being enriched in the FA group. The former can generate the H_2_S and the latter can transform the N_2_ to ammonia gas. Both of H_2_S and ammonia are related to the intestinal diseases based on the previous studies [20]. Therefore, the enrichment of these functional modules in FA group illustrates the deterioration of the gut micro-ecosystem under the FA condition, also implicates that the perturbation of the gut microbiota is correlated with the FA development.

In summary, the present data indicate that sensitization alters the composition of intestinal microbes. The resulted dysbiosis may be related to the pathogenesis of FA.

## MATERIALS AND METHODS

### Animals and samples

All procedures were conducted according to the guidelines for animal care and approved by the ethics committee at Shenzhen University. Conventionally raised 6-8-week-old BALB/c mice were purchased from the Guangdong Experimental Animal Center and housed in a light- and temperature-controlled facility. They were fed with a standard laboratory chow for two weeks and then the control group mice were treated by oral administration of phosphate buffer saline (PBS), and the case group mice were treated by oral administration of allergen in PBS. The composition of the experimental injection is shown in Supplementary Table S1. Throughout the feeding period, feces samples were collected once a week.

### Immune index test

After the trial, we randomly selected 6 mice of control group and 4 mice of FA group, then detect specific IgE, IL-4, IL-5, IL-13 in the mouse serum (Supplementary Table S2) by enzyme-linked immunosorbent assay (ELISA) with reagent kits purchased from the Biomart (Beijing, China) following the manufacturer's instructions.

### Counting the number of mast cells and eosinophils in the intestinal mucosa

A segment of the jejunum was excised from each mouse after the sacrifice and fixed with 4% formalin overnight. Paraffin sections were prepared with the intestinal tissue and stained with 0.5% toluidine blue (for mast cell counts) or hematoxylin & eosin (for eosinophil counts). The number of mast cells and eosinophils was counted in 20 randomly selected fields (×400) under a light microscope. The slides were coded; the observers were not aware of the code to avoid the observer bias.

### Isolation of lamina propria mononuclear cells (LPMC)

The small intestine was excised after the sacrifice, cut into small pieces and incubated with collagenase IV (0.5 mg/ml) at 37 °C for 2 h. The mixed cells were filtered through a cell strainer (40 μm). The LPMC were isolated from the mixed cells by centrifugation with percoll reagent at gradient concentrations. The cell viability was greater than 99% as checked by the Trypan blue exclusion assay.

### Isolation of CD4^+^ CD25^−^ T effector cells (Teff) and dendritic cells (DC)

The Teff cells and DCs were isolated from the LPMCs by magnetic cell sorting (MACS) with commercial reagent kits (Miltenyi Biotech) following the manufacturer's instructions. The cell purity was greater than 98% as checked by flow cytometry.

### Assessment of antigen-specific CD4^+^ T cells

The Teff cells (labeled with CFSE) and DCs were cultured at a ratio of 5:1 in the presence of ovalbumin (OVA; the specific antigen) or bovine serum albumin (BSA; used as an irrelevant antigen) for 3 days. The cells were analyzed by flow cytometry, the CFSE-dilution assay.

### Recording core temperature and diarrhea

After the specific antigen challenge, the core temperature was recorded from each mouse with a digital thermometer at 30 min after the challenge. Diarrhea was recorded within 2 h after the challenge.

### DNA extraction of feces samples

After 5 week trial, fecal samples (up to ~1 g) were collected and immediately transferred to a −20 °C freezer. The total DNA was extracted with the reported methods [21].

### Amplification and sequencing

The 16S rDNA sequencing was performed according to the standard protocols [22] on the Ion PGMTM platform.

### Bioinformatic analysis

The data were treated with in-house pipeline developed based on motor v.1.33.3 [23]. The community structure was calculated based on the membership and relative abundance, based on the proportion of reds, of taxonomic groups in the sample. In addition, we used PICRUSt [13] to produce predicted f KEGG Orthologs (KO) from the 16S rRNA gene sequence data.

### Statistics

Data are presented as mean ± SD. The difference between groups were determined by the Student t test or ANOVA along with the Bonferroni correction if more than 2 groups. P<0.05 was set as a statistically significant criterion.

## SUPPLEMENTARY MATERIALS TABLES
















