# Supplementary material for: Kinetic changes of intestinal microbiota in the course of intestinal sensitization
Source: Oncotarget. 2016 Oct 21;7(49):81197–207. doi: 10.18632/oncotarget.12797 (PMC5348386; doi:10.18632/oncotarget.12797)
Supplement: Supplementary file 1 [file oncotarget-07-81197-s001.pdf]

Kinetic changes of intestinal microbiota in the course of intestinal sensitization

SUPPLEMENTARY TABLES

Supplementary Table S1: Mouse number at each time point throughout the period of immunization FA group. Footnotes: Each mouse was gavage-fed with 1 mg ovalbumin mixed with 20 µg cholera toxin in 0.3 ml PBS at each time point. PBS group: Each mouse was gavage-fed with 0.3 ml saline at each time point.

See Supplementary File 1

Supplementary Table S2: Results of serum IgE, IgG and Th2 cytokines

Footnotes: Ctrl: Control group. FA: Food allergy group.

See Supplementary File 2

Supplementary Table S3: FA-vs-CK, Wilcox-otu

See Supplementary File 3

Supplementary Table S4: FA-vs-CK, Wilcox-KO

See Supplementary File 4

Supplementary Table S5: FA-vs-CK, Wilcox-pathway

See Supplementary File 5

Supplementary Table S6: Cytoscape

See Supplementary File 6

Supplementary Table S7: PBS_FA.PBS.correlation

See Supplementary File 7
